# A case report on the effective and safe use of ravulizumab in atypical hemolytic uremic syndrome during pregnancy

**DOI:** 10.1186/s12882-026-04950-w

**Published:** 2026-04-15

**Authors:** Abdulaziz Alkhaldi, Mamoun Elawad, Sultan Al Dalbhi, Bandar Alquraini, Alanoud Alshannan

**Affiliations:** Prince Sultan Military Medical City, Riyadh, Saudi Arabia

**Keywords:** Atypical hemolytic uremic syndrome, Case report, Eculizumab, Pregnancy, Ravulizumab

## Abstract

**Background:**

Atypical hemolytic uremic Syndrome (aHUS), a form of thrombotic microangiopathy (TMA), had a poor prognosis until the development of complement C5-inhibiting monoclonal antibodies, eculizumab and ravulizumab. While ravulizumab has shown effectiveness in treating postpartum TMA, data about its use during pregnancy remains lacking.

**Case presentation:**

A 32-year-old woman with a history of aHUS was initially diagnosed in 2018 at the age of 27 after presenting with microangiopathic hemolytic anemia, thrombocytopenia, and acute kidney injury (AKI) requiring hemodialysis and plasma exchange. Kidney biopsy showed evidence of TMA, and genetic testing identified a pathogenic CD46 (MCP) variant, confirming complement-mediated aHUS. She achieved complete hematologic and renal recovery with eculizumab therapy and remained dialysis-independent thereafter. Eculizumab was continued until 2021, with brief interruptions during the COVID-19 pandemic, due to intermittent drug shortages. Treatment was discontinued, and the patient remained clinically stable under close monitoring. In 2022, during routine follow-up, she was found to be pregnant without complications. The pregnancy was unplanned; however, she was closely monitored throughout early gestation with regular assessments of renal function, hematologic parameters, blood pressure, proteinuria, and complement activity. Prophylactic complement inhibition was not initiated during early pregnancy because eculizumab was unavailable, and she remained in remission during the first trimester. At 25 weeks’ gestation, she developed a relapse of aHUS characterized by vomiting, diarrhea, anemia, thrombocytopenia, hypertension, and AKI. Stool testing for Shiga toxin–producing Escherichia coli was negative. Eculizumab was unavailable; thus, after informed consent, ravulizumab was initiated based on her body weight as per the manufacturer’s label: a loading dose of 2,700 mg on Day 1 and a maintenance dose of 3,300 mg on Day 14, and subsequently 3300 mg every eight weeks. The patient achieved rapid hematologic and renal recovery and had a spontaneous full-term vaginal delivery without maternal or neonatal complications. Both mother and child remained well after about four years of follow-up on ravulizumab.

**Conclusion:**

This case report highlights the potential role of ravulizumab in managing aHUS during pregnancy. Given the limited safety data, further studies are needed to guide its use in this setting.

## Introduction

Hemolytic Uremic Syndrome (HUS) is a form of thrombotic microangiopathy (TMA) characterized by the triad of microangiopathic hemolytic anemia, thrombocytopenia, and acute kidney injury (AKI) [1]. HUS is broadly classified based on underlying etiology into Shiga toxin-producing Escherichia coli (STEC)-associated HUS and atypical HUS (aHUS). STEC-HUS is typically associated with gastrointestinal infections and represents the most common cause of HUS in children [2]. In contrast, aHUS is a rare, complement-mediated disorder driven by genetic or acquired dysregulation of the alternative complement pathway [3].

The uncontrolled complement activation occurs due to genetic mutations or acquired autoantibodies in the alternative complement pathway [3]. aHUS is a rare, life-threatening disease whose annual incidence is estimated to be 2 cases per million in the United States [4]. It represents the majority of HUS in adults and 5–10% of HUS in children [3]. The global prevalence of aHUS is 4.9 per million people, while the prevalence among patients aged 20 and younger is between 2.2 and 9.4 per million people [5].

The diagnosis of aHUS involves identifying TMA and excluding more common causes such as STEC-HUS and thrombotic thrombocytopenic purpura (TTP), using Shiga toxin testing and ADAMTS13 activity, respectively. aHUS is a diagnosis of exclusion, and complement testing or genetic analysis may support the diagnosis, but are not required for initial treatment [6].

Before the availability of effective treatment, aHUS patients had a very poor prognosis, with up to 78% of the patients either developing kidney failure or dying within a few years [7, 8]. Plasma therapy was the first-line treatment, replacing the non-functioning complement regulators and hyper-functional complement components [3, 7]. Nevertheless, over 50% of aHUS patients undergoing plasma exchange either died or progressed to end-stage kidney disease within three years of the initial event [7]. Since 2011, eculizumab, a humanized monoclonal antibody targeting complement 5 (C5), has effectively blocked complement system overactivation and prevented TMA, significantly improving aHUS outcomes [6]. However, maintenance therapy necessitates frequent intravenous administration, starting with 900 mg weekly for the first four weeks, followed by 1200 mg every two weeks thereafter [6, 9].

To increase the half-life of eculizumab and extend the dosing frequency from 2 to 8 weeks, ravulizumab was derived from eculizumab by changing four amino acids, resulting in optimized pharmacokinetics/pharmacodynamics and a more precise, weight-based dosing regimen. A Phase 3 clinical trial recently reported that treatment with ravulizumab, administered every eight weeks, resulted in rapidly improved hematologic and renal outcomes with no unexpected adverse events in adults with aHUS [10]. While ravulizumab has been shown to treat postpartum TMA from aHUS effectively, there are no publications to date regarding its use during pregnancy in patients with aHUS [11].

With the scarcity of data on ravulizumab use in pregnant women, it is not possible to inform a drug-associated risk of major birth defects, miscarriage, or adverse maternal or fetal outcomes [12]. Additionally, in pregnancy, aHUS is associated with adverse maternal and neonatal outcomes, including maternal and fetal death [13].

Here, we describe the clinical course of a woman with aHUS who experienced disease relapse during pregnancy and was managed with complement inhibition.

## Case presentation

A 32-year-old woman with a history of aHUS was previously diagnosed in December 2018 (at the age of 27) following an episode of microangiopathic hemolytic anemia, thrombocytopenia, and AKI requiring hemodialysis and plasma exchange. Kidney biopsy at that time showed TMA, and genetic testing identified a pathogenic CD46 (MCP) variant. She achieved complete recovery with eculizumab therapy and remained dialysis-independent thereafter under our observation.

In 2018, she first presented with a two-week history of diarrhea associated with nausea, vomiting, and decreased appetite. She had no relevant past medical or surgical history, apart from an uncomplicated spontaneous vaginal delivery six months earlier. She had stable vital signs, normal blood pressure, and her physical examination was unremarkable except for signs of dehydration.

The patient underwent ten sessions of plasmapheresis with 100% replacement using fresh frozen plasma. This was initiated as part of the standard supportive management for suspected TMA following exclusion of TTP (ADAMTS13 activity was within the normal range). Plasma exchange was continued until targeted therapy could be initiated. She also required a whole blood transfusion and initiation of hemodialysis via a tunneled right internal jugular catheter.

In December 2018, the decision was to start eculizumab at a dose of 900 mg every two weeks for the first two doses, and then 1200 mg every two weeks as maintenance. Prior to treatment initiation, plasmapheresis was discontinued, and the patient received meningococcal vaccination, with appropriate antibiotic prophylaxis administered according to institutional protocol. Around the same period, the patient developed severe pulmonary edema requiring mechanical ventilation for one day. This event was attributed to fluid overload related to AKI, hypertension, transfusions, and prior plasmapheresis. She continued eculizumab every two weeks and was closely monitored for hemodialysis requirements. Hemodialysis was discontinued after 4 weeks as kidney function progressively improved and continued to recover until it was normal by six months.

Eculizumab therapy was continued until 2021, with short interruptions during the COVID-19 pandemic due to intermittent drug shortages and difficulties with patient transportation to the infusion center. Treatment was discontinued, and the patient remained clinically stable under close observation to monitor renal functions and the risk of aHUS recurrence during this period off therapy.

Laboratory results (Table 1) demonstrated anemia, thrombocytopenia, elevated lactate dehydrogenase (LDH), and renal impairment, while infectious, autoimmune, and secondary causes were ruled out. The renal ultrasound showed normal-sized kidneys. After the hematology and rheumatology team consultation, she went for a kidney biopsy. Immunological screening and kidney biopsy did not show any evidence suggesting nephritis. The kidney biopsy showed evidence of TMA. Genetic testing performed using whole-exome sequencing (CentoXome GOLD®, CENTOGENE, Germany) identified a homozygous, likely pathogenic variant in the *CD46* gene, NM_172359.2:c.350_351dup, p.(Glu118Thrfs*17), confirmed by Sanger sequencing. This finding established the diagnosis of complement-mediated aHUS.

**Table 1:** Laboratory findings at first presentation, recovery phase, and during relapse in a patient with aHUS

Laboratory investigation	At first presentation		Recovery phase	25-weeks gestation
	Initial investigation	Further investigation		
Haemoglobin (g/L)	90	112	133	86
Platelets (x10^9^/L)	90	40, then 25	192	129
Direct Bilirubin (μmol/L)	8	2	1.5	2
Albumin (g/L)	22	NA	39	30
Urea (mmol/L)	16	NA	8	6.1
Serum creatinine (μmol/L)	560	NA	90	143
Potassium (mmol/L)	4.1	NA	4.4	4.7
Sodium (mmol/L)	136	NA	137	135
Arterial blood gases:				
pH	7.2	NA	NA	NA
Serum bicarbonate (mmol/L)	19	NA	24.9	NA
Partial Pressure of Carbon Dioxide (mmHg)	30	NA	NA	NA
CRP (mg/L)	24	8	NA	13
ESR (mm/hr)	NA	25	24	NA
LDH (U/L)	1376	540	207	364
Haptoglobin (g/L)	<0.058	<0.058	0.31	<0.06
C3 (g/L)	0.62	0.64	1.06	1.02
C4 (g/L)	0.1	0.14	0.16	0.14
ADAMTS13 Activity (N: 0.40–1.30 IU/ml)	1.15	NA	NA	NA
ANA	Negative	Negative	NA	NA
ANCA	Negative	Negative	NA	NA
Glomerular basement membrane antibody	NA	Negative	NA	NA
Complement afctor H (N: 242–759 mg/L)	548	NA	NA	NA
Complement factor I (N: 39.0–100 mg/L)	52.2	NA	NA	NA

Abbreviations: ANA, antinuclear antibody; ANCA, antineutrophil cytoplasmic antibodies; C3, complement component 3; C4, complement component 4; CRP, C-reactive protein; ESR, erythrocyte sedimentation rate; LDH, lactate dehydrogenase; NA, not available

In 2022 (during her routine follow-up visit to the outpatient clinic), the patient was found to be pregnant, despite having previously received counseling that pregnancy could trigger an aHUS relapse. The pregnancy was unplanned; however, she was closely monitored throughout early gestation and the following months. Regular assessments every 3–4 weeks included evaluations of kidney function, blood pressure, complete blood count, hemoglobin level, serum LDH, platelet count, proteinuria, and complement C3 activity. No laboratory or clinical signs of hemolysis, TMA, or kidney injury were detected. Prophylactic complement inhibition was not initiated during early pregnancy, and the patient remained in remission despite the unavailability of eculizumab therapy. The first trimester passed without complications.

At 25 weeks of gestation, she presented with an aHUS relapse characterized by hypertension (blood pressure ~160–170/90–100 mmHg), vomiting, diarrhea, anemia, thrombocytopenia, and AKI (Table 1). Stool testing for STEC was negative. Given the unavailability of eculizumab and the urgency of her clinical condition, the risks and benefits of initiating treatment with ravulizumab, despite the limited experience with it in pregnancy, were discussed with the patient and her husband. Informed consent was obtained, and the patient received appropriate antibiotic prophylaxis. The patient received two doses of ravulizumab based on her body weight as per the manufacturer’s label: a loading dose of 2,700 mg on Day 1, followed by a maintenance dose of 3,300 mg on Day 14, and subsequently 3300 mg every eight weeks.

Follow-up laboratory assessment showed return to baseline levels of serum creatinine, platelet count, and hemoglobin (serum creatinine 120 μmol/L, LDH 169 U/L, platelet count 292 × 10^9^/L, and C3 of 1.24 g/L). The patient had a spontaneous full-term vaginal delivery without any maternal or neonatal complications. In 2026, after about four years of follow-up and regular treatment with ravulizumab, the patient and her child remain in good health without any complications.

**Fig. 1:**
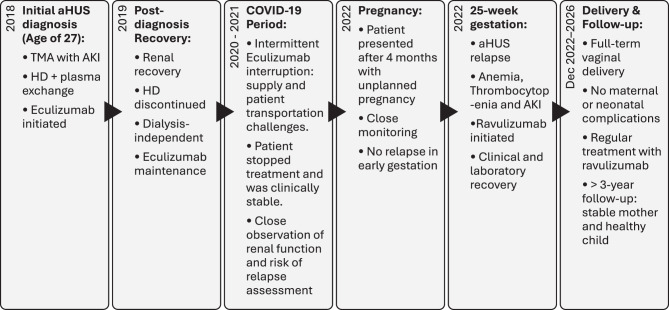
Timeline of diagnosis, treatment, pregnancy, relapse, and follow-up in atypical hemolytic uremic syndrome. Abbreviations: aHUS, atypical hemolytic uremic syndrome; AKI, acute kidney injury; HD, hemodialysis; TMA, thrombotic microangiopathy

The chronological sequence of diagnosis, treatment interruptions, pregnancy, relapse, and follow-up is summarized in Fig. 1.

## Discussion

At the time of initiating ravulizumab therapy, to the best of our knowledge, no published reports described its use throughout pregnancy in patients with aHUS. Our patient, who was treated for aHUS relapse with ravulizumab, started at gestation week 25, gave birth to a healthy infant who has developed normally without any growth abnormalities. Historically, our patient responded well to eculizumab treatment and recovered from the first episode of aHUS flare after six months of therapy. Eculizumab was unavailable, as described above, and the treatment was stopped with close follow-up.

Although the patient’s sustained remission during this period may be attributable to the underlying CD46 (MCP) loss-of-function variant, which in the native kidney has been associated with a comparatively milder phenotype, a lower risk of progression to end-stage kidney disease, and a relatively high rate of relapse. She experienced a clinical relapse during pregnancy after treatment discontinuation. This relapse was characterized by hypertension, diarrhea, proteinuria, and AKI. Prompt initiation of ravulizumab was therefore critical to prevent irreversible renal damage and preserve both maternal and fetal outcomes.

Although eculizumab is considered the standard of care for complement-mediated aHUS during pregnancy, ravulizumab offers comparable efficacy and safety, with the added advantage of a longer dosing interval–every eight weeks instead of every two weeks–improving treatment convenience and adherence. Both agents share the same mechanism of action, targeting complement protein C5, with comparable hematologic and renal outcomes demonstrated in both adult and pediatric populations [14–17]. However, at the time of treatment, no reports were available regarding the use of ravulizumab during pregnancy.

In aHUS, the approved label for ravulizumab recommends a minimum treatment duration of six months, after which continuation should be guided by clinical judgment. While some patients–particularly those who achieve complete hematological and renal remission and have a low-risk genetic profile–may be considered for discontinuation, the risk of recurrence remains. This is especially important in patients of childbearing age, where physiological stressors may trigger disease relapse, underscoring the need for individualized, risk-based treatment decisions. However, our case demonstrates that such discontinuation may not be practical, as it can lead to severe disease recurrence.

The decision not to initiate prophylactic complement inhibition during early pregnancy reflected a combination of clinical stability, close laboratory monitoring, and limited availability of eculizumab rather than an assumption of low relapse risk. At the time, the patient remained in complete hematologic and renal remission, with normal complement levels and no evidence of TMA on serial assessments. However, pregnancy is a recognized trigger for aHUS relapse, and earlier initiation of complement inhibition with eculizumab might have been preferable. This case, therefore, highlights the challenges of balancing maternal-fetal risk, drug availability, and evolving safety data, and underscores that close monitoring alone should not be interpreted as an optimal management strategy in all pregnant patients with aHUS.

Our patient, who had ceased treatment, presented with an aggressive relapse, underscoring the potential risks associated with discontinuation, even in seemingly low-risk individuals [6, 8, 18–20]. Notably, pregnancy is a well-recognized trigger for TMA, particularly in patients with aHUS [21]; this highlights the need for vigilant, long-term monitoring–especially during high-risk periods such as pregnancy. Ravulizumab has shown remarkable efficacy in the management of the aHUS flare during the second and third trimesters of pregnancy. The patient recovered completely from aHUS flare, as evidenced by normalized serum creatinine, platelets, and hemoglobin levels. Moreover, the patient delivered a spontaneous vaginal delivery without any complications. After about four years of regular ravulizumab treatment (3.25 years after delivery), the mother has not developed any complications, and her child has not demonstrated any signs of growth defects. This report contributed valuable clinical insight into the management of relapsed aHUS during pregnancy, demonstrating that ravulizumab initiated at relapse was associated with hematologic and renal recovery, allowing for the discontinuation of hemodialysis in a patient with prior successful treatment with eculizumab.

Pregnancy for women with aHUS was associated with a greater risk of adverse maternal and neonatal outcomes, including maternal and fetal death [13]. Although data on the use of eculizumab in pregnancy remain limited, recent real-world evidence has shown its safety and efficacy in pregnant women with aHUS, both as prophylactic and curative therapy, even in those requiring dialysis or who have undergone transplantation [22].

Complement inhibition during pregnancy raises important considerations regarding fetal exposure. At the time of treatment, data regarding placental transfer of ravulizumab were not available. Subsequent evidence has demonstrated that ravulizumab can cross the placenta, as shown in a recent case report documenting detectable drug levels in cord blood [23] Importantly, emerging clinical data support its safety in pregnancy: a recent multicenter analysis of 19 pregnancies in patients with paroxysmal nocturnal hemoglobinuria treated with ravulizumab reported favorable maternal and fetal outcomes, with all pregnancies resulting in live births and no reported congenital abnormalities. These findings provide reassurance regarding the use of ravulizumab during pregnancy, although additional data are needed to further define its safety profile in this setting [24]. Nonetheless, eculizumab remains the preferred therapy during pregnancy until additional safety data on ravulizumab become available [25]. Although clinical data on the use of ravulizumab in pregnant women with aHUS are lacking [12], this case adds to the limited but growing evidence suggesting its potential safety and effectiveness for both mother and child.The key strengths of this report are its unique approach and encouraging outcome. This is among the first case studies to document the use of ravulizumab for aHUS during pregnancy and more than three years after delivery, with both the mother and child remaining healthy. Our report is limited because it is based on only one case, so it cannot be expanded to a larger population. In addition, data about the ravulizumab concentrations in the blood and breast milk of the mother, as well as in the blood of her infant, are unavailable. Such information should be gathered in future cases to better inform about the safety of using ravulizumab during pregnancy. Long-term data would be beneficial as well.

## Conclusion

This case report adds to a growing body of evidence showing the potential benefits of ravulizumab treatment for pregnant patients with aHUS. It might contribute to expanding the limited therapeutic options in aHUS pregnant cases. Real-world evidence for safety during pregnancy is limited owing to its recent approval; clinical studies in aHUS pregnant cases, including a close follow-up, would be beneficial to guide clinical practice.

## Data Availability

The identified CD46 variant has been submitted to the ClinVar database under accession number SCV007496431.

## Abbreviations

aHUS: Atypical HUS
AKI: Acute kidney injury
ANA: Antinuclear antibody
ANCA: Antineutrophil cytoplasmic antibodies
C3: Complement 3
C4: Complement 4
C5: Complement 5
CRP: C-reactive protein
ESR: Erythrocyte sedimentation rate
HUS: STEC: Shiga toxin-producing Escherichia coli
LDH: Lactate dehydrogenase
STEC-HUS: Shiga toxin-producing Escherichia coli-associated Hemolytic Uremic Syndrome
TMA: Thrombotic microangiopathy
TTP: Thrombocytopenic purpura
